# Supplementary material for: Macrophage Activation Assays to Evaluate the Immunostimulatory Capacity of Avibacterium paragallinarum in A Multivalent Poultry Vaccine
Source: Vaccines (Basel). 2020 Nov 10;8(4):671. doi: 10.3390/vaccines8040671 (PMC7712920; doi:10.3390/vaccines8040671)
Supplement: Supplementary file 1 [file vaccines-08-00671-s001.pdf]

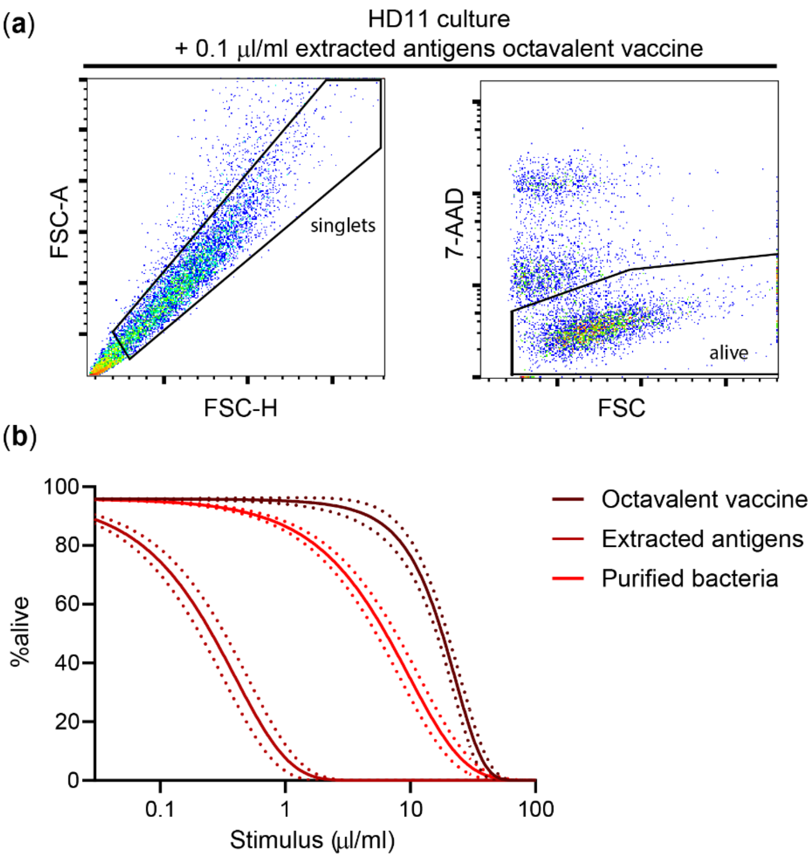

Supplementary Figure S1. Cytotoxicity of the octavalent vaccine, extracted antigens and purified *Av. paragallinarum* bacteria. (**a**) The HD11 cells were stained with 7-aminoactinomycin D to determine the percentage of viable cells by flow cytometry. The cells were first gated for single cells, also excluding debris. Next, cells negative for 7-AAD were considered to be alive. (**b**) HD11 cells were stimulated with graded doses of octavalent vaccine (0.3 – 30 µl/ml), the antigens extracted from the octavalent vaccine (0.1 – 10 µl/ml) and purified *Av. paragallinarum* bacteria (0.1 – 10 µl/ml) for 48 h. The graph was created by plotting survival curves with confidence intervals.

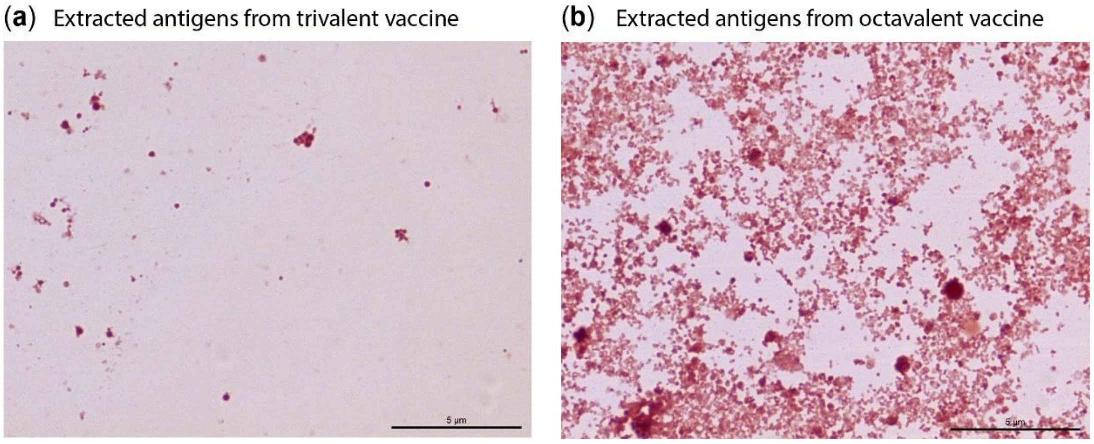

Supplementary Figure S2. Gram staining of extracted antigens from the tri- (**a**) and octavalent (**b**) vaccines. *Av. paragallinarum* bacteria were purified from the octavalent vaccine as described in the Materials and Methods. As a control, the antigens from the trivalent vaccines were purified using the same procedure. The images were taken at 1000x magnification using an Olympus BX60 microscope and immersion oil. The scale bars in black represent 5 µm.

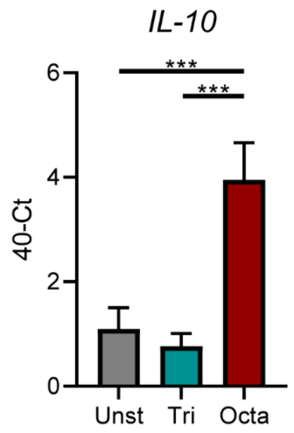

Supplementary Figure S3. The octavalent vaccine induced gene expression of *IL-10*, whereas a trivalent vaccine without Av. paragallinarum antigens did not. Gene expression levels of *IL-10* by HD11 cells were determined 8 h after stimulation with 1.0 µl/ml tri- or octavalent vaccine and are expressed as 40-Ct values, as described by Eldaghayes et al. [34]. The figure shows three independent technical replicates. The error bars represent the SEM. A Kruskal-Wallis test combined with Dunn's multiple comparisons test was used to test for statistical significance of the data. *** $p < 0.001$.
